# A Novel Nitrite Reductase from *Acinetobacter haemolyticus* for Efficient Degradation of Nitrite

**DOI:** 10.3390/biom15010063

**Published:** 2025-01-04

**Authors:** Xiao-Yan Yin, Emmanuel Mintah Bonku, Jian-Feng Yuan, Zhong-Hua Yang

**Affiliations:** 1 Xingzhi College, Zhejiang Normal University, Jinhua 321100, China; yinxiaoyan@zjnu.edu.cn (X.-Y.Y.); jf_yuan@zjnu.edu.cn (J.-F.Y.); 2 State Key Laboratory of Drug Research, Shanghai Institute of Materia Medica, Chinese Academy of Sciences, Shanghai 201203, China; emmanuelbonku@simm.ac.cn

**Keywords:** nitrite reductase, *Acinetobacter haemolyticus*, nitrite reduction, homology modeling

## Abstract

Nitrite reductases play a crucial role in the nitrogen cycle, demonstrating significant potential for applications in the food industry and environmental remediation, particularly for nitrite degradation and detection. In this study, we identified a novel nitrite reductase (*Ah*NiR) from a newly isolated denitrifying bacterium, *Acinetobacter haemolyticus* YD01. We constructed a heterologous expression system using *E. coli* BL21/pET28a-*Ah*Nir, which exhibited remarkable nitrite reductase enzyme activity of 29 U/mL in the culture broth, substantially higher than that reported for other strains. Structural analysis of *Ah*NiR revealed the presence of [Fe-S] clusters, with molecular docking studies identifying Tyr-282 and Ala-289 as key catalytic sites. The enzymatic properties of *Ah*NiR demonstrated an optimal pH of 7.5 and an optimal catalytic temperature of 30 °C. Its kinetic parameters, K_m_ and *v*_max,_ were 1.53 mmol/L and 10.18 mmol/min, respectively, fitting with the Michaelis–Menten equation. This study represents the first report of a nitrite reductase from a denitrifying bacterium, providing a new enzyme source for nitrite degradation applications in the food industry and environmental remediation, as well as for biosensing technologies aimed at nitrite detection.

## 1. Introduction

Nitrite is an important nitrogenous compound in the natural nitrogen cycle. Its accumulation in the biosphere has increased due to excessive use of nitrogen fertilizers [1]. Additionally, it is commonly added to food products to enhance color and preservation, particularly in the production of salted foods such as pickled vegetables, cured meats, and pickled fish. These salted foods are particularly popular in East Asia, notably in countries like China and South Korea. However, the excessive nitrite levels in food pose serious threats to human health [2,3]. Furthermore, the accumulation of nitrite in the natural environment presents significant public health concerns [4]. Thus, it is crucial to develop effective methods for the removal of excessive nitrite from both food and the environment.

Biocatalysis technology has emerged as a feasible solution for nitrite degradation due to its numerous advantages, such as high efficiency, environmental friendliness, and low cost [5,6]. In recent years, nitrite reductase (NiR), a key enzyme for nitrite degradation, has garnered increasing attention from researchers in the fields of enzymology. NiR shows promising applications in food processing, environmental remediation of nitrite pollution, and the development of biosensors for nitrite detection [7,8]. In processes such as cheese and sausage production, nitrite reductases play a vital role in controlling the levels of residual nitrites, thereby ensuring food safety and quality.

NiRs are mainly found in microorganisms, including bacteria and microalgae, and they have also been identified in some plant species [9,10]. There are four major categories of NiRs, namely CuNiR, cd1NiR, ccNiR, and FdNiR, as reported in previous studies [6,10,11,12]. NiRs from different organisms typically exhibit distinct enzymatic properties; for example, NirK from *Halorussus* sp. YCN54 is known to be halophilic and slightly thermophilic [13], while NirK from *Bacillus firmus* Gy-49 demonstrates notable stability and strong resistance to organic solvents [14]. Although several NiRs have been reported in previous studies, the development of novel NiRs with good catalytic performance and application potential remains a critical focus in the field of enzyme research.

In the biological denitrification process, NiR is essential for nitrite reduction. Generally, highly active denitrifying bacteria contain abundant NiRs [6]. In our previous work, we isolated a strain of bacteria with high denitrification capacity from the activated sludge of a coking wastewater treatment plant. Preliminary results indicated that this bacterium exhibited robust nitrite reduction abilities. In this work, we report the characterization of a novel nitrite reductase with outstanding catalytic performance derived from this denitrifying bacterium. This represents the first report of a NiR from denitrifying bacteria, showcasing promising application prospects for nitrite degradation in both the food industry and environmental pollutant treatment, as well as serving as a sensing element for biosensors aimed at nitrite detection.

## 2. Materials and Methods

### 2.1. Materials

Tryptone and yeast extract were purchased from Oxoid Limited (Hampshire, UK). DNA and plasmid extraction kit was procured from Guangzhou Magen Biotechnology Co., Ltd. (Guangzhou, China). The tool enzyme for gene manipulation, including restriction endonucleases and ligases, was purchased from New England Biolabs Inc. Sodium nitrite (AR grade) was purchased from Sinopharm Chemical Reagent Co., Ltd. (Shanghai, China). Other reagents not specifically mentioned were purchased from Aladdin^®^ Biochemical Technology Co., Ltd. (Shanghai, China) and Sinopharm Chemical Reagent Co., Ltd., all of AR grade. The denitrifying bacterium was isolated and preserved at −80 °C in our laboratory. The plasmid pET28a was used as the cloning and expression vector, while *E. coli* BL21 (DE3) served as the host cell for NiR expression. The microbial strains were preserved at −80 °C in our laboratory.

### 2.2. Identification of the NiR-Producing Bacterium

The isolated denitrifying bacterium was identified using molecular biology techniques, specifically by 16SrDNA sequencing, along with physiological and biochemical tests [15]. The isolated denitrifying bacterium was inoculated on a BTB medium plate containing Na_2_HPO_4_·7H_2_O (7.9 g), MgSO_4_·7H_2_O (1.0 g), sodium succinate (4.7 g), NaNO_2_ (0.72 g), and KH_2_PO_4_ (1.5 g) in 1000 mL H_2_O, supplemented with 0.1% bromothymol blue as an indicator and solidified with 1.4% agar [16]. A single colony exhibiting a blue transparent circle was selected and further incubated in a BTB liquid medium. The genomic DNA of the collected bacterial cells was extracted with the DNA extraction kit, and the 16SrDNA was sequenced by Sangon Biotech (Shanghai, China) Co., Ltd. (shown in Supplementary Materials, Figure S1). The 16SrDNA sequence was submitted to NCBI for BLAST analysis, and homologous sequences were downloaded to construct a phylogenetic tree based on the 16SrDNA sequences using MEGA 7.0 [17]. The physiological and biochemical tests were also conducted following the guidelines presented in Bergey’s Manual of Determinative Bacteriology [18] to further confirm the strains.

### 2.3. Construction of NiR Heterologous Expression System

The genome of the isolated denitrifying bacterium, identified as *Acinetobacter haemolyticus,* was analyzed to identify the gene encoding the putative nitrite reductase through gene mining technology (DNA sequence shown in Supplementary Materials, Figure S2) [15]. The putative *Ah*NiR gene was amplified via PCR using the following primers: *Ah*Nir-F: 5′-CAGCAAATGGGTCGCG/GATCCATGTATTTATATACTGATTTCGATCAACAAC-3′ and *Ah*Nir-R: 5′-GTGGTGGTGGTGGTGC/TCGAGTTAAGCATAAGCACGCTCCTTAAA-3′, incorporating *Bam*H I and *Xho* I restriction enzyme sites (underlined). The PCR product underwent digestion with *Bam*H I and *Xho* I before being purified and cloned into pET28a, followed by transformation into *E. coli* BL21 (DE3) to construct the NiR heterologous expression system *E. coli* BL21 (DE3)/pET28a-*Ah*Nir (as shown in Supplementary Materials, Figure S3). The expression of *Ah*NiR was induced by adding IPTG to a final concentration of 0.1 mmol/L when the culture reached an optical density at 600 nm (OD600) of 0.6, with induction carried out for 20 h at 30 °C.

### 2.4. Purification of the Recombinant Protein

Induced recombinant cells were harvested by centrifugation at 15,000× *g* for 10 min and washed twice with PBS buffer at pH 7.0. The collected cells were resuspended in PBS buffer containing 1 mM PMSF and disrupted using an ultrasonic cell breaker. The supernatant of the cell lysate was obtained by centrifugation at 22,000× *g* for 10 min at 4 °C, followed by filtration through a 0.22 μm filter membrane to yield the *Ah*NiR crude enzyme solution, which was subsequently analyzed by SDS-PAGE. The crude enzyme was loaded onto a Ni-NTA Purose 6 Fast Flow column (QIAGEN, Shanghai, China) equilibrated with ten column volumes of a buffer consisting of 20 mM Tris-HCl (pH 7.4) containing 1 mM PMSF. The loaded crude enzyme sample was washed with five-column volumes of Tris-HCl buffer containing 20 mmol/L imidazole before eluting the protein with Tris-HCl buffer containing 250 mmol/L imidazole. Collected fractions containing *Ah*NiR were analyzed using SDS–PAGE.

### 2.5. AhNiR Enzymatic and Catalytic Properties

The enzymatic properties of *Ah*NiR were investigated by assessing its response to temperature, pH, and metal ions, along with its stability and kinetic parameters. Kinetic properties of *Ah*NiR were determined under optimum temperature and pH conditions with NaNO_2_ concentrations ranging from 0.51 to 2.17 mmol/L. The Michaelis–Menten model was employed to simulate the kinetic equation, and kinetic parameters, K_m_ and v_max_ of *Ah*NiR were calculated using nonlinear fitting based on the least squares method performed via the Levenberg–Marquardt algorithm in Origin (OriginPro 2021 9.8.0.200).

The thermal stability of *Ah*NiR was assessed by incubating it at a series of temperatures (20, 25, 30, 35, 40, and 50 °C) for 1 h, followed by the determination of the residual activity. The pH stability was evaluated by incubating samples in buffers of various pH (from 5.0 to 11.0) for 2 h, followed by a determination of the residual activity.

### 2.6. Structural Modeling and Catalytic Simulation

The enzyme’s three-dimensional structure is essential for elucidating its catalytic mechanism. The three-dimensional structure of *Ah*NiR was calculated by homology modeling via the Swiss-Model workspace without a signal peptide, employing NirA from *Mycobacterium tuberculosis* (PDB: 1ZJ9) as a template [19].

To explore the molecular mechanism of *Ah*NiR catalysis during nitrite reduction, molecular docking analysis between *Ah*NiR and NO_2_^−^ was performed using AutoDock software (4.2.6) to simulate ligand–receptor interactions through genetic algorithms. PyMOL software (3.1.3) facilitated the visualization of results to identify binding sites and hydrogen bonds between *Ah*Nir and NO_2_^−^.

### 2.7. Analytical Techniques

NiR activity was assessed using an improved method developed by Martinez-Espinosa et al. [20], which is based on the colorimetric determination of nitrite. The assay mixture contained a final volume of 200 μL comprising 40 μL PBS solution at pH 7.0, 20 μL of Na_2_S_2_O_4_ (0.01 mol/L), 10 μL each of NaCl (0.01 mol/L) and methyl viologen (MV: also at 0.01 mol/L), along with 20 µL of nitrite substrate solution (14.5 mol/L) and 100 μL of enzyme solution; samples were incubated at 30 °C. The reaction was initiated by adding dithionite (DT) solution at 30 °C for 10 min and stopped by the oxidation of DT through vigorous stirring. The disappearance of nitrite was determined after a 40-fold dilution of 25 μL of the reaction mixture using the diazo coupling method with naphthalene ethylenediamine hydrochloride as the chromogenic agent, analyzed by spectrophotometry at 538 nm [21]. NiR activity is defined as the amount of enzyme required to consume 1 μmol of NaNO_2_ per minute and is referred to as one NiR activity unit (U).

## 3. Results and Discussion

### 3.1. Identification of Strains

**Figure 1:**
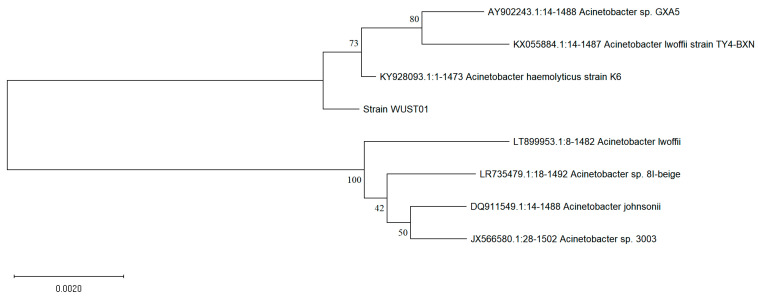
Phylogenetic tree of A. haemolyticus YD01 based on 16SrDNA gene sequence and other related sequences.

The nitrite degradation ability of the strain grown on BTB was assessed, yielding a degradation rate of 32.75 mg/(L·h), significantly higher than that of *Streptomyces mediolani*’s EM-B2, which exhibited a rate of 2.01 mg/(L·h) [22]. The colony morphology was characterized by smooth white spots with distinct edges, and the strain was identified as a Gram-negative bacterium. The obtained 16SrDNA sequence (shown in Supplementary Materials Figure S1) was submitted to NCBI for BLAST analysis. A phylogenetic tree was constructed using MEGA 5.0 software with the Neighbor-Joining algorithm, as shown in Figure 1.

The results confirmed that this strain belongs to the genus *Acinetobacter*. Subsequently, physiological and biochemical tests were conducted following Bergey’s Manual of Determinative Bacteriology (as shown in Supplementary Materials, Table S1) [18]. The colony morphology was similar to that of *Acinetobacter* sp. FYF8 [23]. Based on these findings, this bacterium was designated as *Acinetobacter haemolyticus* YD01. Previous studies have demonstrated that *Acinetobacter* species possess the ability to degrade nitrite [23,24,25], yet the specific nitrite reductase responsible for this activity had not been identified or reported until recently. Isolating this enzyme and elucidating its catalytic mechanism are essential for understanding its denitrification mechanism and enhancing its efficiency. Importantly, this provides a new high-performance enzyme for nitrite reduction applications.

### 3.2. Construction of AhNiR Heterologous Expression System and Expression

**Figure 2:**
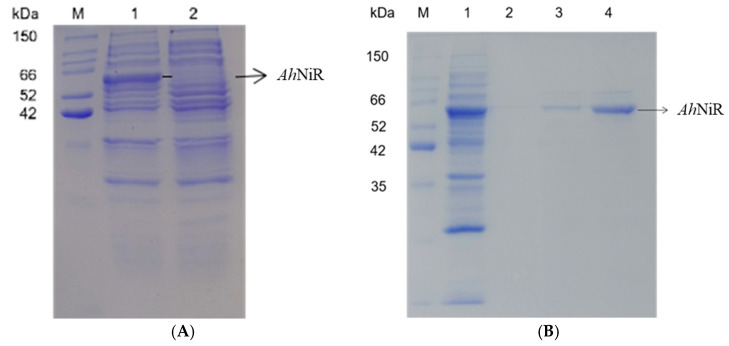
SDS-PAGE of heterologous expression *Ah*NiR. (**A**) Expressed *Ah*NiR in *E. coli* BL21 (Lane M: Protein Marker; Lane 1: *E. coli* BL21/pET28a-*Ah*NiR; Lane 2: *E. coli* BL21/pET28a); (**B**) Purified *Ah*NiR by Ni-NTA Purose 6 Fast Flow column affinity chromatography (Lane M: Protein Marker; Lane 1: *Ah*NiR crude enzyme solution; Lane 2: Tube 1 eluent; Lane 3: Tube 2 eluent; Lane 4: Tube 3 eluent).

The *Ah*NiR gene (sequence presented in Supplementary Materials, Figure S2) was amplified via PCR using genomic DNA *A. haemolyticus* YD01 as the template. The PCR product was digested with *Bam*H I and *Xho* I before being cloned into the pET28a vector (schematic diagram shown in Supplementary Materials, Figure S3). Confirmation of pET28a-*Ah*NiR was achieved by double-enzyme digestion, as depicted in the agarose electrophoresis shown in Supplementary Materials, Figure S4. The recombinant plasmid pET28a-*Ah*NiR was successfully transformed into *E. coli* BL21 (DE3), resulting in the establishment of *E. coli* BL21 (DE3)/pET28a-*Ah*NiR. *Ah*NiR expression was induced using IPTG, yielding an enzyme activity of 29 U per milliliter culture broth. The heterologously expressed *Ah*NiR was purified by Ni-NTA Purose 6 Fast Flow column affinity chromatography, achieving a specific enzyme activity of 127 U/mg_protein_. The expressed and purified products were analyzed by SDS-PAGE, as presented in Figure 2.

The target band corresponding to approximately 61 kDa was observed in samples from *E. coli* BL21/pET28a-*Ah*NiR induced with IPTG; however, no band was present in the control (*E. coli* BL21/pET28a). This target band size aligned with the predicted molecular mass of *Ah*NiR (61929 Da), calculated based on the amino acid sequence (shown in Supplementary Materials, Figure S5).

### 3.3. Enzymatic Properties and Catalytic Properties of AhNiR

**Figure 3:**
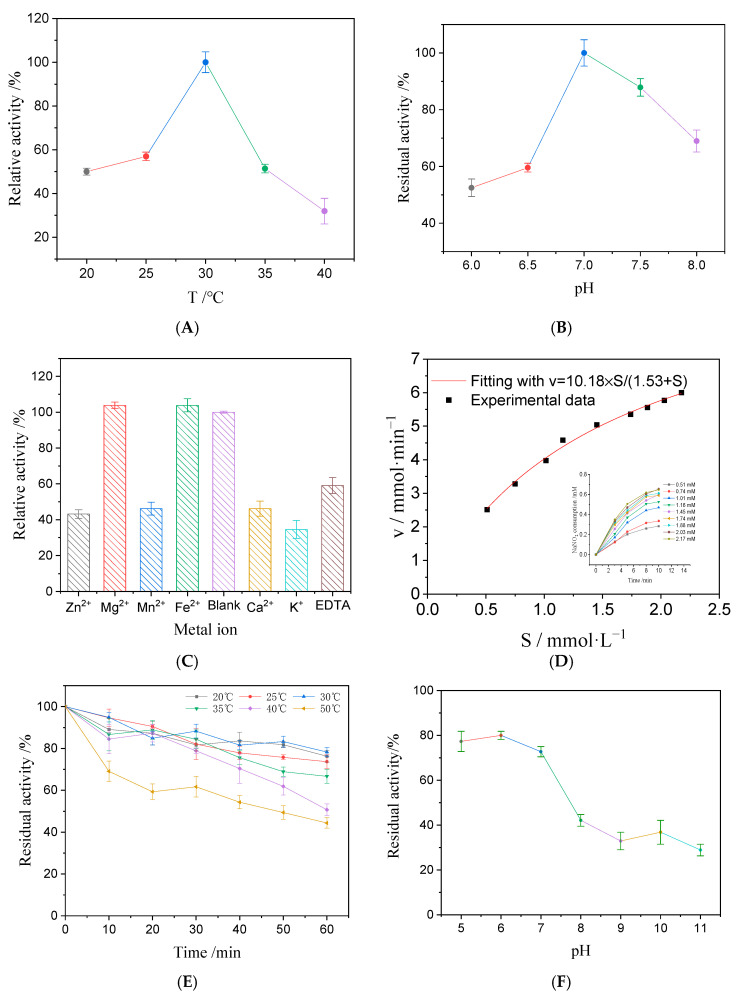
*Ah*NiR enzymatic properties. (**A**) Effect of reaction temperature on the *Ah*NiR activity; (**B**) effect of reaction pH on the *Ah*NiR activity; (**C**) effect of ion on the *Ah*NiR activity; (**D**) Kinetic parameter simulation of *Ah*NiR; (**E**) thermal stability; (**F**) pH stability.

The enzymatic and catalytic properties of *Ah*NiR were investigated and evaluated, including optimal pH and temperature, metal ion effects, reaction kinetics, and stability assessments. The results are presented in Figure 3.

*Ah*NiR demonstrated a broad activity range between 20 °C and 40 °C (Figure 3A), with maximum activity observed at 30 °C while retaining over 50% activity between 25 °C and 35 °C. The optimal catalysis temperature of *Ah*NiR is lower than that reported for some other NiRs, such as NirK_Hrs_ from *Halorussus* sp., which operated at 55 °C [13], and LJ01-NiR from *Bacillus cereus,* which operated at 35 °C [26]. This indicates that *Ah*NiR can achieve effective catalytic activity at relatively low reaction temperatures, thus helping maintain enzyme activity and avoiding thermal inactivation. The optimal pH for *Ah*NiR activity was determined to be 7, with approximately 90% relative activity retained at pH 7.5 (Figure 3B). This suggests that the enzyme favors weak alkaline conditions, which aligns with the viewpoint of Fan and Xia, indicating that similar enzymes exhibit higher activity under such conditions [23,24]. To assess the influence of metal ions on *Ah*NiR activity, several ions, Zn^2+^, Mg^2+^, Fe^2+^, Mn^2+^, Ca^2+^, and K^+,^ were selected and, respectively, tested and added onto the enzyme reaction system alongside EDTA as a metal ion chelating agent. Both Mg^2+^ and Fe^2+^ slightly enhanced *Ah*NiR activity (Figure 3C), a characteristic also observed in NiR from *Bacillus firmus* Gy-49 [14]. Structural evaluations suggest that *Ah*NiR may belong to the cd1NiR that facilitates intermediate formation, leading to the release of nitric oxide. Conversely, Zn^2+^, Mn^2+^, Ca^2+^, and K^+^ exhibited inhibitory effects on *Ah*NiR; notably, Zn^2+^ inhibition has also been documented for NiRs from other bacteria such as *Pseudomonas stutzeri* PSN-1 [27] and *Bacillus cereus* LJ01 [26].

The reaction kinetics were fitted to the Michaelis–Menten equation; the nonlinear fitting curve is presented in Figure 3D. The v_max_ was calculated at 10.18 mmol/min, with a K_m_ value of 1.53 mmol/L. This K_m_ value is comparable to that reported for LJ01-NiR from *Bacillus cereus* LJ01 [26], while other studies have indicated K_m_ values of 4.0 mmol/L for NiR from *Hfx. mediterranei* [28] and 3.2 mmol/L for NirK_Hrs_ from *Halorussus* sp. YCN54 [13]. Compared to these values, *Ah*NiR demonstrates a superior affinity for nitrite, suggesting a promising application for *Ah*NiR for nitrite reduction. Both thermal stability and pH stability were assessed; results are presented in Figure 3E,F, respectively. Overall, *Ah*NiR exhibited moderate thermal stability at room temperature, with good stability observed over time; after an hour of incubation at 50 °C, approximately 45% residual enzyme activity remained (Figure 3E). Regarding pH stability (Figure 3F), *Ah*NiR retained about 80% activity after 2 h incubation at pH 6.0 but experienced rapid loss of function under alkaline conditions.

### 3.4. Structural Evaluation of AhNiR

**Figure 4:**
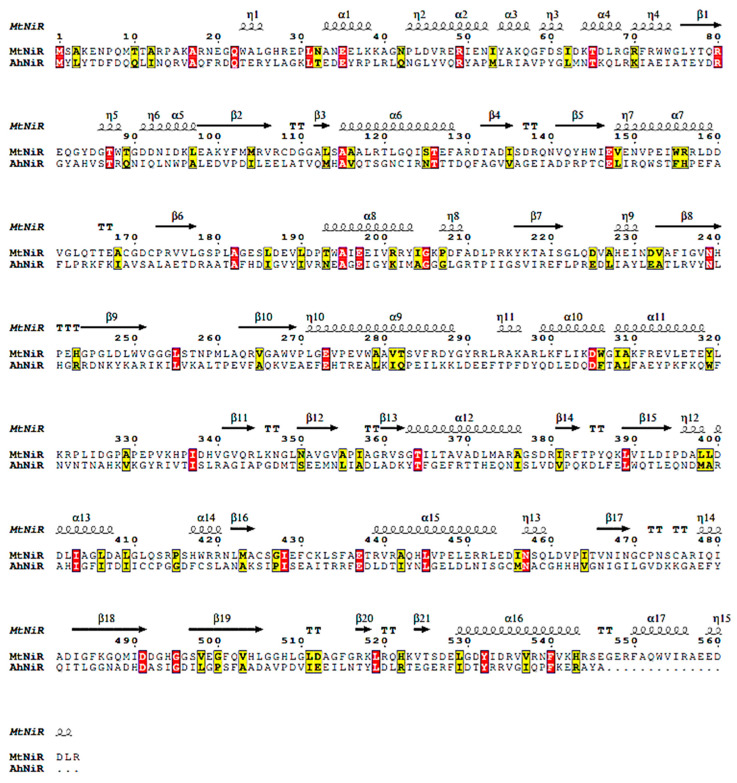
Sequence alignment and secondary structure analysis of *Ah*NiR and *Mt*NiR.

Copper-type NiRs (CuNiRs) are prevalent among microbial species, and each CuNiR typically contains six Cu^2+^ [29]. To determine whether *Ah*NiR contains Cu^2+^ ions or not, it was treated with DDTC (sodium diethyldithiocarbamate, Cu^2+^ chelating agent). The results (presented in Supplementary Materials, Table S2) showed that Cu^2+^ removal did not adversely affect *Ah*NiR activity, suggesting that *Ah*NiR does not contain Cu^2+^. BLAST analysis of the amino acid sequence revealed high homology between *Ah*NiR and a NiR from *Mycobacterium tuberculosis* (PDB: 1ZJ9), which contains two [Fe-S] clusters and a heme-binding domain. The amino acid sequence alignment and secondary structure analysis are presented in Figure 4.

**Figure 5:**
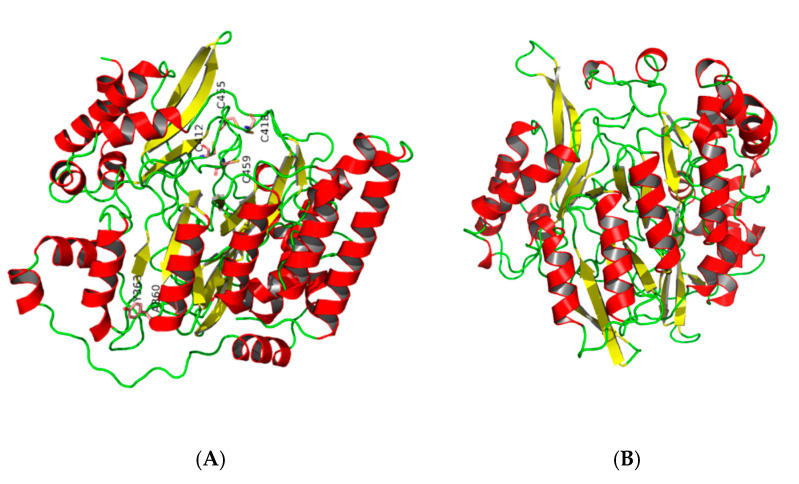
*Ah*NiR structure by homologous modeling. (**A**) *Ah*NiR and (**B**) 1Zj9.

The structural model of *Ah*NiR was built using *Mt*NiR (nirA) (PDB: 1ZJ9) from *Mycobacterium tuberculosis* [19] as a template, which has a known NiR crystal structure. The simulated results are presented in Figure 5.

**Figure 6:**
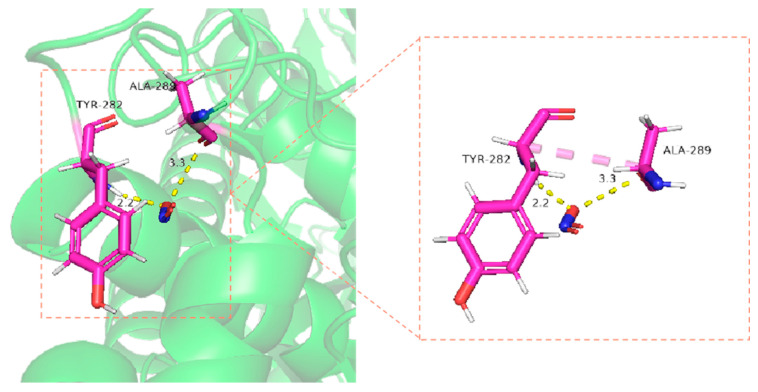
Model of interaction between *Ah*NiR and substrate NO_2_^−.^

The reliability of the structural *Ah*NiR simulation was evaluated using ProCheck server analysis. The results are shown in Supplementary Materials, Figure S6a. The evaluation results indicated that 84.2% of residues fell within optimal regions while an additional 15.8% resided within the allowed regions. This suggests that the protein model is structurally reasonable. Additionally, surface potential analysis of *Ah*NiR (presented in Supplementary Materials, Figure S6b) revealed substantial positive potential areas on the protein surface conducive to substrate binding with NO_2_^−^. Molecular docking analysis further elucidated interactions between *Ah*NiR and NO_2_^−^; results indicated substrate binding sites at Ala289 and Tyr282 where hydrogen bonds formed with lengths measuring approximately 3.3 Å and 2.2 Å, respectively (Figure 6).

## 4. Conclusions

For the first time, a novel *Ah*NiR was obtained from a newly isolated denitrifying bacterium, A. haemolyticus YD01. Structural analysis revealed that *Ah*NiR shares a similar structure with *Mt*NiR (NirA) from *Mycobacterium tuberculosis*, based on both amino acid sequence alignment and 3D structural modeling. *Ah*NiR demonstrated remarkable nitrite reduction activity at room temperature and neutral pH conditions, with kinetic parameters showing a v_max_ of 10.18 mmol/min and K_m_ of 1.53 mmol/L, respectively. These findings indicate that *Ah*NiR possesses significant catalytic activity and a strong nitrite affinity, suggesting promising applications for nitrite degradation in the food industry or environmental pollutant treatment and as a sensing element in biosensors for nitrite detection. To enhance the practical applications of *Ah*NiR, our future research will focus on improving the enzyme’s catalytic efficiency via rational design strategies, as well as conducting comprehensive investigations into its nitrite reduction mechanism. This approach aims to optimize the enzyme’s performance and unlock its full potential for diverse biotechnological and environmental applications.

## Data Availability

Data are contained within the article and Supplementary Materials.

## Supplementary Materials

The following supporting information can be downloaded at: https://www.mdpi.com/article/10.3390/biom15010063/s1, Table S1 Physiological and biochemical tests for the isolated nitrite-degrading bacteria; Table S2: Effect of DDTC (diethyldithiocarbamate) on the AhNiR activity; Figure S1: 16SrDNA sequence of the isolated denitrifying bacteria; Figure S2: The DNA sequence of AhNiR; Figure S3: Schematic diagram of the construction of the recombinant plasmid pET28a-AhNiR; Figure S4: Agarose electrophoresis for recombinant plasmid with double-enzyme digestion; Figure S5: Amino acid sequence of AhNiR. Figure S6 Reliability assessment of protein simulations and surface charge analysis of AhNiR.
