# Supplementary material for: A Novel Nitrite Reductase from Acinetobacter haemolyticus for Efficient Degradation of Nitrite
Source: Biomolecules. 2025 Jan 4;15(1):63. doi: 10.3390/biom15010063 (PMC11764342; doi:10.3390/biom15010063)

Supplementary material

A novel nitrite reductase from *Acinetobacter haemolyticus* for efficient degradation of nitrite

Xiao-Yan Yin[1], Emmanuel Mintah Bonku[2], Jian-feng Yuan[1], Zhong-Hua Yang[1]*

[1]Xingzhi College, Zhejiang Normal University, Jinhua 321100, China

[2]State Key Laboratory of Drug Research, Shanghai Institute of Materia Medica, Chinese Academy of Sciences, Shanghai 201203, China

* Corresponding author

Zhong-Hua Yang, ORCID: 0000-0001-8341-4775

E-mail: yangzh@zjnu.edu.cn or yangzju@163.com

STable 1 Physiological and biochemical test for the isolated nitrite degrading bacteria

Test item	Results
Nitrate	+
CITR	+
Catalase	+
Oxidase	-
Phenylalanine dehydrogenase	-
Gelatin	+

Note: “+” positive result; “-” Negative result

STable 2 Effect of DDTC (diethyldithiocarbamate) on the *Ah*NiR activity

Group	Enzyme activity (U/mL)
DDTC+*Ah*NiR	7±0.33
PBS+ *Ah*NiR	7±0.16

Test conditions: 2 mL of the crude enzyme solution was mixed with 1 mL of 0.01 mol/L DDTC solution and incubated. The control was incubated with phosphate buffer. Enzyme activity was detected at 30°C.

SFig. 1 16SrDNA sequence of the isolated denitrifying bacteria

1 GGCTCAGATT GAACGCTGGC GGCAGGCTTA ACACATGCAA GTCGAGCGGG

51 GAAGTGTAGC TTGCTACATT ACCTAGCGGC GGACGGGTGA GTAATGCTTA

101 GGAATCTGCC TATTAGTGGG GGACAACATT CCGAAAGGAA TGCTAATACC

151 GCATACGTCC TACGGGAGAA AGCAGGGGAT CTTCGGACCT TGCGCTAATA

201 GATGAGCCTA AGTCGGATTA GCTAGTTGGT GGGGTAAAGG CCTACCAAGG

251 CGACGATCTG TAGCGGGTCT GAGAGGATGA TCCGCCACAC TGGGACTGAG

301 ACACGGCCCA GACTCCTACG GGAGGCAGCA GTGGGGAATA TTGGACAATG

351 GGCGGAAGCC TGATCCAGCC ATGCCGCGTG TGTGAAGAAG GCCTTTTGGT

401 TGTAAAGCAC TTTAAGCGAG GAGGAGGCTA CTCTAGTTAA TACCTAGAGA

451 TAGTGGACGT TACTCGCAGA ATAAGCACCG GCTAACTCTG TGCCAGCAGC

501 CGCGGTAATA CAGAGGGTGC GAGCGTTAAT CGGATTTACT GGGCGTAAAG

551 CGTGCGTAGG CGGCTGATTA AGTCGGATGT GAAATCCCTG AGCTTAACTT

601 AGGAATTGCA TTCGATACTG GTCAGCTAGA GTATGGGAGA GGATGGTAGA

651 ATTCCAGGTG TAGCGGTGAA ATGCGTAGAG ATCTGGAGGA ATACCGATGG

701 CGAAGGCAGC CATCTGGCCT AATACTGACG CTGAGGTACG AAAGCATGGG

751 GAGCAAACAG GATTAGATAC CCTGGTAGTC CATGCCGTAA ACGATGTCTA

801 CTAGCCGTTG GGGCCTTTGA GGCTTTAGTG GCGCAGCTAA CGCGATAAGT

851 AGACCGCCTG GGGAGTACGG TCGCAAGACT AAAACTCAAA TGAATTGACG

901 GGGGCCCGCA CAAGCGGTGG AGCATGTGGT TTAATTCGAT GCAACGCGAA

951 GAACCTTACC TGGTCTTGAC ATAGTAAGAA CTTTCCAGAG ATGGATTGGT

1001 GCCTTCGGGA ACTTACATAC AGGTGCTGCA TGGCTGTCGT CAGCTCGTGT

1051 CGTGAGATGT TGGGTTAAGT CCCGCAACGA GCGCAACCCT TTTCCTTATT

1101 TGCCAGCGGG TTAAGCCGGG AACTTTAAGG ATACTGCCAG TGACAAACTG

1151 GAGGAAGGCG GGGACGACGT CAAGTCATCA TGGCCCTTAC GACCAGGGCT

1201 ACACACGTGC TACAATGGTC GGTACAAAGG GTTGCTACCT AGCGATAGGA

1251 TGCTAATCTC AAAAAGCCGA TCGTAGTCCG GATTGGAGTC TGCAACTCGA

1301 CTCCATGAAG TCGGAATCGC TAGTAATCGC GGATCAGAAT GCCGCGGTGA

1351 ATACGTTCCC GGGCCTTGTA CACACCGCCC GTCACACCAT GGGAGTTTGT

1401 TGCACCAGAA GTAGGTAGTC TAACCGTAAG GAGGACGCTT ACCACGGTGT

1451 GGCCGATGAC TGGGGTGAAG TCGTA

SFig. 2 The DNA sequence of *Ah*NiR

1 ATGTATTTAT ATACTGATTT CGATCAACAA CTGATTAATC AACGTGTTGC

51 TCAGTTCCGT GATCAAACAG AACGCTATTT AGCGGGAAAA TTGACTGAAG

101 ACGAGTACCG TCCTTTACGT TTGCAAAATG GTTTATATGT GCAACGTTAC

151 GCACCTATGT TGCGTATTGC CGTGCCGTAT GGCTTGATGA ATACAAAACA

201 ACTTCGAAAA ATTGCTGAGA TTGCGACTGA GTATGATCGT GGATATGCAC

251 ACGTATCGAC TCGTCAAAAC ATCCAGTTGA ATTGGCCTGC ATTGGAAGAT

301 GTGCCAGATA TTCTTGAGGA ATTGGCAACA GTTCAAATGC ACGCAGTTCA

351 AACATCTGGT AACTGTATTC GTAATACGAC AACAGATCAG TTTGCTGGCG

401 TTGTTGCGGG TGAGATTGCT GATCCACGTC CAACATGCGA ATTGATTCGT

451 CAATGGAGTA CATTCCATCC AGAATTTGCA TTCTTACCAC GTAAATTCAA

501 AATTGCAGTT TCTGCACTGG CTGAAACAGA CCGTGCCGCT ACAGCATTCC

551 ATGATATTGG CGTGTATATC GTGCGTAACG AGGCGGGTGA AATAGGCTAT

601 AAAATCATGG CTGGTGGGGG CTTGGGTCGT ACCCCAATCA TTGGTAGTGT

651 CATTCGTGAG TTCTTACCGC GTGAAGATTT AATTGCTTAT CTTGAAGCGA

701 CTTTGCGTGT TTATAATTTG CATGGTCGCC GTGATAACAA ATATAAAGCA

751 CGTATTAAAA TTCTCGTAAA AGCATTAACG CCTGAAGTAT TCGCACAAAA

801 GGTTGAAGCT GAATTTGAAC ACACACGTGA AGCTTTGAAG ATTCAGCCTG

851 AAATCTTGAA AAAGTTAGAT GAAGAATTTA CCCCATTTGA TTATCAAGAT

901 TTAGAAGATC AAGATTTTAC GGCATTATTT GCTGAATATC CTAAATTTAA

951 GCAATGGTTC AATGTCAATA CCAATGCGCA TAAAGTCAAA GGCTATCGTA

1001 TCGTGACGAT TTCCTTGAAG CGTGCGGGTA TTGCACCGGG TGATATGACG

1051 TCAGAGGAAA TGAACTTAAT TGCAGACTTA GCTGATAAAT ATACTTTTGG

1101 CGAATTCCGC ACCACACACG AACAGAATAT TTCGTTAGTG GATGTGCCTC

1151 AAAAAGATTT GTTTGAGTTG TGGCAAACAC TTGAACAAAA CGATATGGCT

1201 CGCGCACATA TCGGTTTTAT CACGGATATT ATTTGCTGTC CTGGCGGAGA

1251 TTTCTGTTCA TTGGCAAATG CTAAATCTAT TCCAATTTCA GAGGCGATCA

1301 CCCGTCGTTT TGAAGACTTG GATACCATCT ATAACTTAGG TGAGCTAGAT

1351 TTAAATATCT CGGGTTGTAT GAATGCATGT GGTCATCACC ATGTCGGAAA

1401 TATTGGTATT CTCGGTGTAG ATAAAAAAGG TGCTGAGTTC TATCAAATCA

1451 CATTGGGCGG TAATGCAGAT CATGACGCTT CAATTGGTGA CATTTTAGGA

1501 CCATCTTTTG CGGCGGACGC AGTGCCCGAT GTTATTGAAG AAATTTTGAA

1551 TACTTACCTC GATCTACGTA CAGAGGGTGA GCGTTTCATT GACACATATC

1601 GCCGTGTTGG TATTCAACCA TTTAAGGAGC GTGCTTATGC TTAA

SFig. 3 Schematic diagram of the construction of the recombinant plasmid pET28a-*Ah*NiR

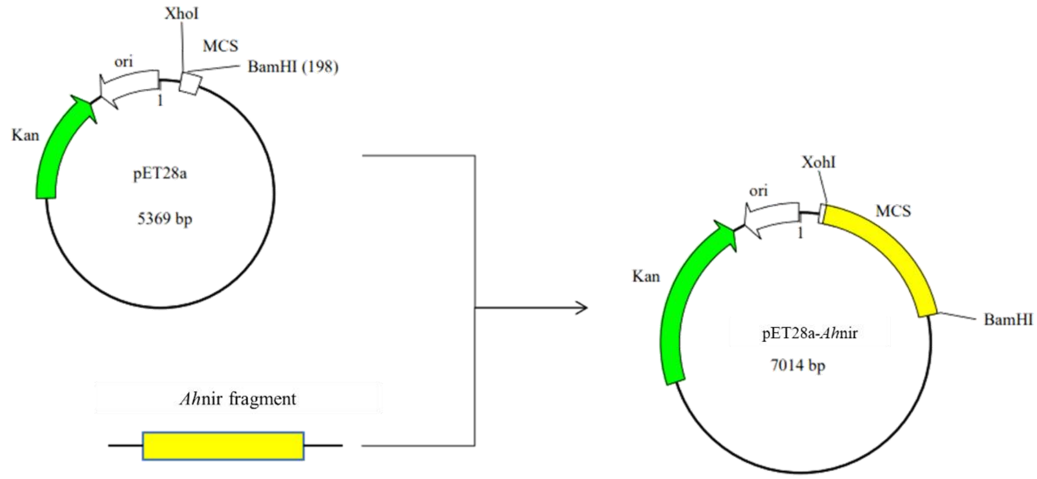

SFig. 4 Agarose electrophoresis for recombinant plasmid with double-enzyme digestion

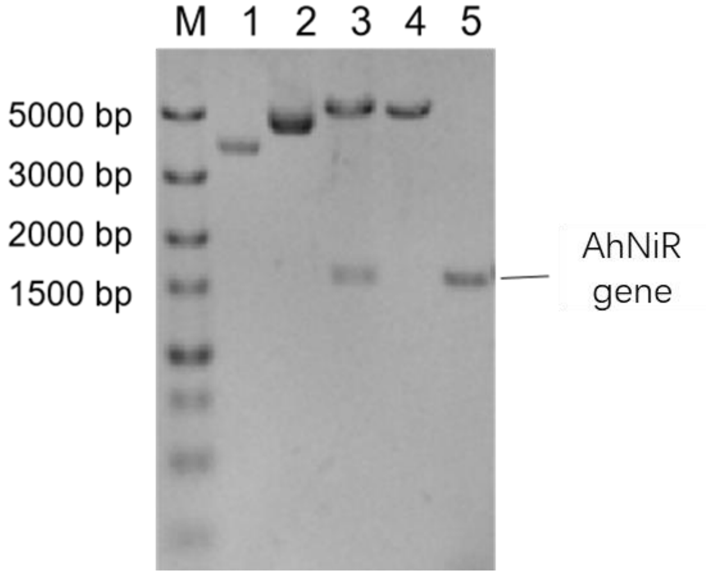

Lane M: DNA ladder; Lane 1: pET28a; Lane 2: pET28a-*Ah*NiR; Lane 3: double-enzyme digestion of pET28a-*Ah*NiR; Lane 4: Double-enzyme digestion of pET28a; Lane 5: PCR product use pET28a-*Ah*NiR as the template

SFig. 5 Amino acid sequence of *Ah*NiR

```
1       MYLYTDFDQQ LINQRVAQFR DQTERYLAGK LTEDEYRPLR LQNGLYVQRY
51      APMLRIAVPY GLMNTKQLRK IAEIATEYDR GYAHVSTRQN IQLNWPALED
101     VPDILEELAT VQMHAVQTSG NCIRNTTTDQ FAGVVAGEIA DPRPTCELIR
151     QWSTFHPEFA FLPRKFKIAV SALAETDRAA TAFHDIGVYI VRNEAGEIGY
201     KIMAGGGLGR TPIIGSVIRE FLPREDLIAY LEATLRVYNL HGRRDNKYKA
251     RIKILVKALT PEVFAQKVEA EFEHTREALK IQPEILKKLD EEFTPFDYQD
301     LEDQDFTALF AEYPKFKQWF NVNTNAHKVK GYRIVTISLR AGIAPGDMTS
351     EEMNLIADLA DKYTFGEFRT THEQNISLVD VPQKDLFELW QTLEQNDMAR
401     AHIGFITDII CCPGGDFCSL ANAKSIPISE AITRRFEDLD TIYNLGELDL
451     NISGCMNACG HHHVGNIGIL GVDKKGAEFY QITLGGNADH DASIGDILGP
501     SFAADAVPDV IEEILNTYLD LRTEGERFID TYRRVGIQPF KERAYA
```

SFig. 6 Reliability assessment of protein simulations and surface charge analysis of *Ah*NiR

A: Ramachandran plot of *Ah*NiR model; B: Surface potential distribution of *Ah*NiR protein

A

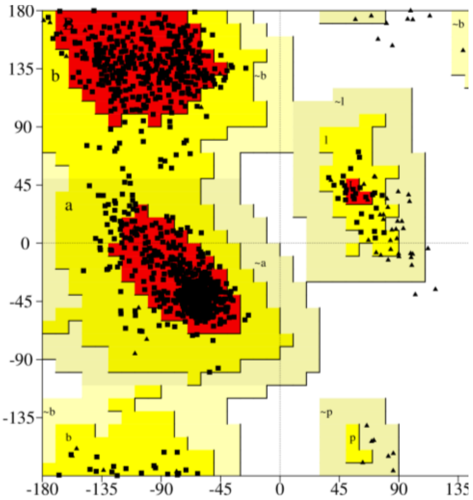

B

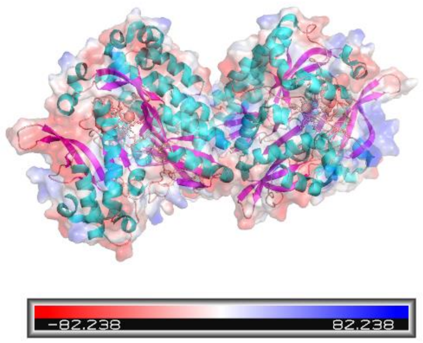

Supplement: Supplementary file 1 [file biomolecules-15-00063-s001.zip › biomolecules-3364968-supplementary.pdf]
